# Calomel in Large Doses as a Diuretic in Dropsies

**Published:** 1851-12

**Authors:** Wm. H. McKee

**Affiliations:** Raleigh, North Carolina


### *Calomel in large doses as a Diuretic in Dropsies.* By WM. H. McKEE, M. D., of Raleigh, North Carolina.

In the treatment of dropsies, calomel has been considered from its earliest introduction into use, as a valuable adjuvant in combination with other remedies, but in no single instance am I aware that it is mentioned by writers as acting as a diuretic when given alone either in small or large doses. Its use in large doses is no new practice, for it has been given freely in the treatment of fever and cholera, and for its hydragogue, cathartic, sialogogue and deobstruent properties, it is well known. In combination with squills, nitre, and sometimes digitalis, it is a very popular mode of administration, and one that generally fulfils the indication for which it is prescribed; but there are cases in which it fails as well as the rest of the class of diuretics, and the physician is thrown back on his own resources for something more active and reliable. When such is the case, calomel in doses from forty to a hundred grains repeated for two or three nights successively, will certainly fulfil the most sanguine hopes, by not only freely purging, but by producing copious diuresis, and at the same time sedation, followed by sleep such as cannot be had from opium nor its preparations, with safety. During its action, gin or whiskey toddy may be freely used, should there be much prostration; but this is seldom the case, the stimulus only aiding the diuretic action. I have seen persons who were unable to lie down, for a week at a time, longer than a few moments, sleep well after the action of the first dose.

The usual mode of administration is to give fifty grains of calomel for three successive nights, and should it fail to produce the desired effect, to wait for several days and repeat the calomel in eighty or a hundred grain doses, but in no instance that has come under my observation, have I ever known the fifty grain doses fail; and in but one case have I ever given a hundred grains. This was followed by free and copious purging at first, and in a short time by diuresis. It is not often that the first dose will act as a diuretic, but after the administration of the second its effects will be produced; the third dose I have never known to fail. During its action, it will, in some instances, produce violent emesis, and sometimes act as an emeto-cathartic; but this only facilitates its action, and no serious consequence may be apprehended. While using these doses, vinegar should be freely applied to the mouth and throat, so as to prevent its specific effect, and cold drinks of all kinds prohibited. Calomel given in the above doses is no more liable to produce its constitutional effect, than it is, when given in smaller ones. I have known one grain to produce salivation without accomplishing any good, but evidently harm. When the indication requires it, it should be used freely. As with quinine, we may produce ringing in the ears; and many contend that when this is the case, the specific effect is produced, and all is accomplished that may be expected of it. But this is known to be false. In the treatment of fever it is continued until the fever is subdued, without any detriment to the patient. So it is with the calomel in large doses in the treatment of dropsies. By using the necessary precaution, you can succeed in introducing into the system such quantities as will fulfil the indication, with no more danger than there is in giving smaller ones. When salivation is produced, it is as manageable as under any other form, and yields as promptly to the usual remedies. I do not recommend its use in these doses in all cases of dropsies, for many of them are merely functional, and the sequelæ of some acute disease, and readily yield to milder remedies; but it is in those cases where there may be hepatic and splenic derangement, when the ordinary remedies fail, that calomel may be relied upon, not only for its cathartic properties, but mainly for its diuretic action. So prompt is it in producing this effect, that I have known it to succeed in expelling the fluid where there was organic disease of both the heart and lungs. If the constitution of the patient is very delicate, for instance a female, doses of thirty to forty grains will be large enough. I was called to see a lady about fifty years of age, who had been pronounced by her medical attendant to be in the last stage of hydrothorax; with cough; bloody expectoration; great dyspnœa; inability to lie down; general anasarca and distension of the abdomen; countenance anxious; tongue red, pointed and dry. She had taken a great variety of remedies, and appeared to be hourly growing worse. I prescribed thirty grains of calomel to be aided by whiskey toddy, and to be repeated the second night. The first dose produced copious purging, the second acted as an emetic with moderate purging, but with profuse diuresis. On visiting her the third day after administering the first dose, she was so much relieved that she was enabled to lie down and sleep with comfort, and to get up and walk without assistance. Her cough and expectoration ceased; tongue became moist, and all difficulty of breathing disappeared. But it is just to remark that the specific effect of the calomel was freely developed, but yielded promptly to the ordinary mode of treatment. After this, she was treated with chalybeates and the preparations of quinine, and recovered her usual health within a few weeks.

The diet in all cases should be very abstemious. After the action of the calomel, it will be necessary to administer some remedies to prevent a re-accumulation of the fluid. I have found a solution of the muriated tincture of iron to answer the indication very well, but in some instances it will disagree with the stomach. When such is the case, the tartrate of iron and potassa suspended in wine—about an ounce to the quart, and a dessert spoonful given three times a day—will suit the most delicate stomachs, as well as fulfil the indication desired. Should the bowels become constipated, and require an aperient or cathartic, cream of tartar and epsom salts equally combined will answer the purpose. But should the re-accumulation of water take place in spite of these means, it will become necessary to repeat the calomel, and follow it by the same treatment. In a number of instances I have found the following combination to answer the purpose when most remedies had disappointed me, especially when the calomel was contra-indicated from constitutional causes. Take one pint of the tincture of asclepias syriaca, which is made by adding one ounce of the fresh root to one pint of whiskey ; let it stand for fourteen days, and then add it to two pints of the strong decoction of sarsaparilla, with one drachm of powdered alum. Of this, give a tablespoonful three or four times a day. Should it not be active enough, or too active, it may be regulated according to the ability of the patient to bear it. It will keep the bowels gently moved two or three times a day, besides acting as a good diuretic and tonic, increasing the appetite and strength.

Too much attention cannot be paid to the manner of dressing as well as the diet. Flannel should always be worn next the skin, and exposure of all kind avoided, especially in damp and rainy weather ; for after the fluid has been expelled from the system, the muscular and cellular tissue is so much relaxed, that if these precautions are not attended to, and aided by proper bandaging of the abdomen, the fluid will, as a matter of course, re-accumulate. By adhering to this, the tissues are aided as they gradually assume their natural healthy and tonic action, and are enabled to resist the morbid influence ; for, in fact, this is the great difficulty in curing hydropic diseases.

In confirmation of my own experience in the diuretic action of calomel, the following extracts from the published proceedings of the Medical Society of the State of North Carolina, held in May last, will give additional testimony to its efficacy.

A discussion arose as to the *modus operandi* of calomel, on an enquiry made by Dr. J. E. Williamson of Caswell, if calomel was in fact a stimulus, or what was the received opinion as to its action ? and he referred to an instance where it had been administered in very large doses by a friend, with decided benefit, in dropsy, acting as a sedative and diuretic at the same time.

Dr. Charles E. Johnson, of Raleigh, "remarked that he had considerable experience in the exhibition of both drugs (calomel and quinine) in large doses, and could testify to their sedative value and influence when given in this manner. Calomel, he believed, in doses of 50, 100, or more grs., particularly in some cases of dropsies, depending upon enlargement of the liver and spleen, and obstruction of the portal circulation, was followed by the happiest results, producing copious and free discharges, sometimes very watery, but not always corresponding in quantity with the increase of the doses, which was, perhaps, generally true of increasing doses of calomel; and that in many instances, sometimes after the second dose, more generally after the third, when the doses have been given day after day, it acted *powerfully as a diuretic.* He had seen many cases of dropsy of this kind improve rapidly, and speedily recover under this treatment. He had never seen salivation produced in this manner, although the action of the calomel was decidedly *sedative*, inducing quiet and sleep, diminished frequency of the heart's action, and an improved state of the secretions generally, with a moist tongue and skin, and all without much, if any prostration."

I am aware that it is too often the case that a general conclusion is drawn from a mere isolated fact, but such is not the case in the present communication. I could give a number of cases to illustrate the above treatment, but deem it unnecessary, as the general principle is here laid down. The main object in directing the attention of the profession to this subject at all, is, that they may see what is the experience of others, and to show that calomel possesses a property far more reliable than has generally been conceded to it as a diuretic, and surpasses all other remedies of that class in dropsical diseases. Should the experience of others, after having given it a fair trial, accord with my own, I shall feel that my humble labors have not been in vain.

